# Four complete mitochondrial genomes of living wild-type chinese giant salamander *Andrias davidianus* (Amphibia: Cryptobranchidae)

**DOI:** 10.1080/23802359.2018.1524275

**Published:** 2018-10-27

**Authors:** Jing-Cheng Xu, Li-Fang Peng, Yan-Rong Chen, Dian-Cheng Yang, Qi-Neng Wu, Shi-Yang Weng, Yong Zhang, Song Huang

**Affiliations:** a College of Life and Environment Sciences, Huangshan University, Huangshan, China;; b School of Sciences, Tibet University, Lahsa, China;; c Xiuning Institute of Rare Aquatic Animals, Huangshan, China

**Keywords:** Mitogenome, endangered species, true genetic background, phylogenetics

## Abstract

The Chinese giant salamander (CGS), *Andrias davidianus* (Amphibian, Caudata, Cryptobranchidae), is endemic to China. After overhunting in the 1990’s, it is very difficult to find the CGS in the wild. Due to mating disorder, the captive breeding population is genetically confounded. The genetic backgrounds of all wild-release individuals in China are not explicit. Herein, we reported four living wild-type complete mitochondrial genomes of this species. The gene order and contents are identical to those found in typical vertebrates. Thirteen protein-coding genes (PCGs) of 7 *A*. *davidianus* (4 from this study, 3 retrieved from GenBank) and 11 other closely species retrieved from GenBank were used to reconstruct phylogenetic tree. The Maximum likelihood (ML) topology shown that the clade of CGS has two subclades with a high support (100%). This study provides partial fundamental information for further exploring the true genetic background of whole population of *A*. *davidianus*.

The family Cryptobranchidae contains two genera: *Cryptobranchus* in North America and *Andrias* in China and Japan. *Andrias* includes two species: *A*. *davidianus* in China and *A*. *japonicus* in Japan. The CGS is the largest extant amphibian in the world (Fei et al. 2006), which is endemic to China. Due to overhunting and habitat loss, the population size of *A*. *davidianus* is very small and still dwindling in the wild. The molecular genetic research of the captive breeding CGSs did not present an obvious geographical pattern (Murphy et al. 2000; Zhang et al. 2002; Wang et al. 2004; Tao et al. 2005, 2006). Due to mating disorder, the captive breeding population is genetically confounded. The genetic backgrounds of all wild-release individuals in China are not explicit.

Three complete mitogenome sequences of this species had been reported (Zhang et al. 2003; Feng et al. 2016; Xu et al. 2016). Four living wild-type *A*. *davidianus* (provenances knew, Voucher number: HS16092-5) have been feeding in Xiuning Institute of Rare Aquatic Animals, Huangshan, Anhui with Barcode Chip Markers. HS16092 (Barcode Chip Markers 900111880167149, weight 12.19 kg, total length 1.30 m) was collected from Huaqiao village, Xiuning County, Huangshan City, Anhui Province, China, in the summer of 1994. HS16093 (900111880167147, 13.13 kg, 1.40 m) was collected from Zhangjiajie City, Hunan Province, China in 2002. HS16094 (900111880167148, 7.50 kg, 0.80 m) was got from Jingzhou City, Hubei Province, China in 2006. HS16095 (900111880167150, 13.12 kg, 1.40 m) was sampled from Jinzhai County, Lu’an, City, Anhui Province, China in 2008.

The four tissue samples were collected by noninvasive methods, including oral epithelium and exfoliating skin. All tissue samples were preserved in 95% ethanol for subsequent DNA extraction and deposited at the Museum of Huangshan University (HUMA20170001-4).

The total length of the mitogenome (GenBank Accession KX298239-42) of HS16094 (HS16092, HS16093, HS16095) was 16 569 (16 567, 16 568, 16 499) bp, and the base composition was 31.9% for A, 32.7% for T, 14.4% for G and 21.0% for C. The four mitogenomes all consist of 13 typical vertebrate protein-coding genes (PCGs), 22 tRNA genes, 2 rRNA genes (12S and 16S rRNA), and 1 control regions (D-loop). Their gene order, contents, and base composition are identical to those found in typical vertebrates.

Three mitogenome sequences of CGS and 11 mitogenome sequences of closely-related species were retrieved from GenBank. We deposited thirteen PCGs of 7 *A*. *davidianus* (4 from this study, 3 retrieved from GenBank) and 11 other closely-related species retrieved from GenBank together as a concatenated supergene for each species. We aligned all these genes using Clustal X (Thompson et al. 1997). Maximum likelihood method was used to reconstruct phylogenetic trees (Figure 1) in http://www.phylo.org/portal2/login!input.action. The phylogenetic analysis result was consistent with the previous research with a high support.

**Figure 1.**
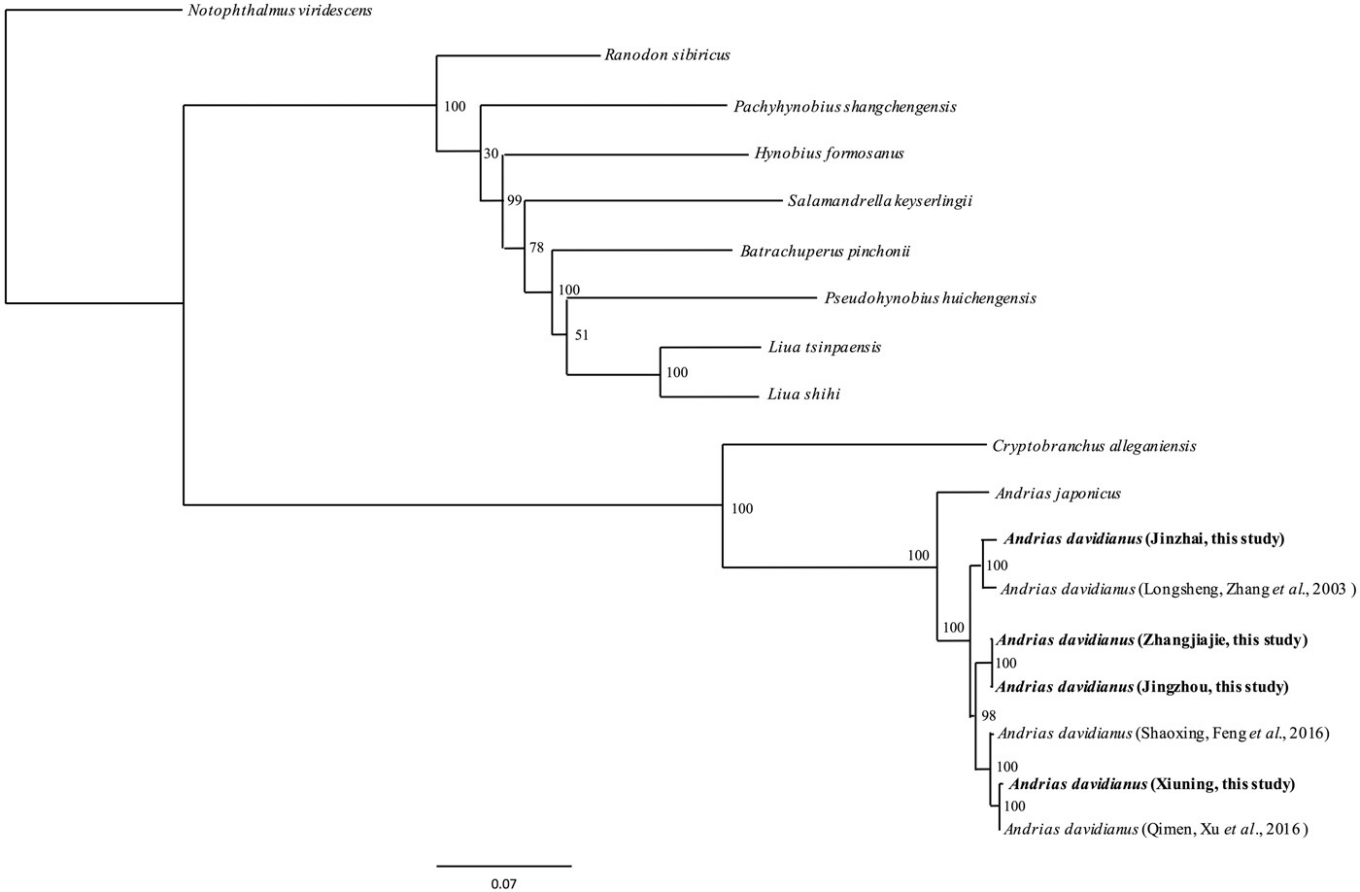
A ML tree of 12 species was constructed based on the dataset of 18 concatenated supergenes by online tool RAxML. The numbers above the branch meant bootstrap value. Bold black highlighted this study species. Sequence data used in the study from GenBank are the following: *Andrias japonicas* (AB208679), *Andrias davidianus* (KX268733, KT119359, AJ492192), *Notophthalmus viridescens* (EU880323), *Cryptobranchus alleganiensis* (GQ368662), *Pachyhynobius shangchengensis* (DQ333812), *Salamandrella keyserlingii* (JX508762), *Pseudohynobius huichengensis* (FJ532060), *Liua shihi* (DQ333810), *Liua tsinpaensis* (KP233806), *Batrachuperus pinchonii* (KP122337), *Hynobius formosanus* (DQ333816), *Ranodon sibiricus* (AJ419960).

The CGS was once widely distributed in the middle and lower drainages of the Yangtze, Yellow and Pearl Rivers, including the 18 Provinces in China (Zhang et al. 2002; Wang et al. 2004). It once had a large population size before the 1980’s. In the early 1990’s, due to its high economic value, the CGS had been overhunted. The number of CGS in the wild has dramatically reduced and still dwindling. This species was listed as the second class of protection of China. From 1990’s many Chinese aquafarms have begun feeding CGS. Wild-release of the CGS in successive years have begun from earlier of this century.

In order to prevent inbreeding depression and outbreeding depression, protect genetic diversity, guide parental mating and wild-release, it is very important and urgent to understand the true genetic background of whole population of *A*. *davidianus*, therefore to guarantee the genetic health of breeding population and wild population.
